# Mechanical Thrombectomy for Acute Pulmonary Embolism in Non-Operating Room Anesthesia (NORA) Locations: Best Safety Practices and Local Insights

**DOI:** 10.3390/healthcare13030227

**Published:** 2025-01-23

**Authors:** Omar Elmadhoun, Jeffrey Huang, Arnoley S. Abcejo, Michael P. Merren

**Affiliations:** 1 Division of Anesthesia and Critical Care Medicine, Mayo Clinic, Rochester, MN 55902, USA; merren.michael@mayo.edu; 2 Department of Anesthesiology and Perioperative Medicine, Mayo Clinic, Rochester, MN 55905, USA; huang.jeffrey@mayo.edu (J.H.); abcejo.arnoley@mayo.edu (A.S.A.)

**Keywords:** pulmonary embolism, mechanical thrombectomy, catheter directed thrombolytic, PERT, non-operating room anesthesia (NORA)

## Abstract

Mortality rates from pulmonary embolism (PE) remain significant, highlighting the need for alternative treatment strategies beyond traditional anticoagulation. Percutaneous interventions, including mechanical thrombectomy and catheter-directed thrombolysis, are emerging as promising options. Given the complex pathophysiology and unique risk profiles of these patients, meticulous multidisciplinary planning is essential. Anesthesiologists play a central role in coordinating care and managing perioperative risks to improve outcomes. This article provides insights into best safety practices and shares experiences from a leading quaternary center. It offers guidance for anesthesia providers to proactively engage in comprehensive risk stratification, participate in multidisciplinary discussions, and support robust contingency planning for managing PE patients undergoing percutaneous interventions in non-operating room anesthesia settings.

## 1. Introduction

**Figure 1:**
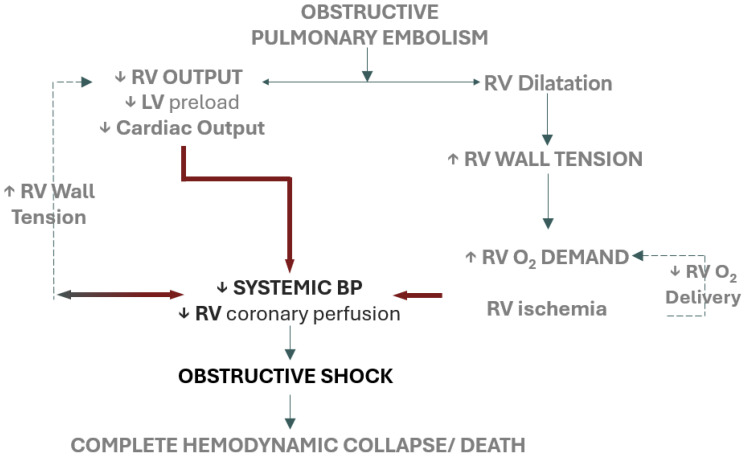
Spiral of events leading to obstructive shock and death in acute pulmonary embolism (PE): Acute PE increases right ventricular (RV) afterload, leading to RV dilation, elevated RV wall tension, and increased oxygen demand. This sequence leads to RV ischemia, reduced contractility, decreased left ventricular (LV) preload, and lowered cardiac output, ultimately reducing RV coronary perfusion pressure and progressing to obstructive shock and death.

Mortality from high-risk pulmonary embolism (PE) in the United States has been increasing over the past two decades [1]. Most PEs begin as thrombi in the deep veins of the calves which then detach and travel through the systemic venous circulation and right-sided heart chambers before lodging in the pulmonary arteries. Morbidity from PE is driven by a complex pathophysiology involving increased pulmonary vascular resistance (PVR) due to mechanical obstruction and release of vasoconstrictive agents such as serotonin, thromboxane-A2, thrombin, histamine, and endothelin, ultimately resulting in right ventricular failure [2]. If not identified or treated promptly, this condition can progress to obstructive shock and ultimately result in death (Figure 1). Due to this rising mortality, patients with high-risk PE who have failed or have contraindications to medical therapy may benefit from interventional therapies. Open surgical thromboembolectomy is an invasive procedure that typically requires sternotomy, cardiopulmonary bypass, circulatory arrest, and excision of the pulmonary artery [3]. As such, percutaneous interventions such as catheter-directed thrombolysis (CDT) and percutaneous mechanical thrombectomy (MT) have emerged as promising alternatives [4]. MT can produce rapid reperfusion without the need for thrombolytic therapy, thus reducing the risk of bleeding that complicates alternative options. While there is limited data comparing outcomes with CDT to other therapies [5], recent data from the FLASH and PERT registries suggest a significant improvement in both 30-day mortality and 30-day major bleeding events (0.8%/1.4%) with MT [6] compared to anticoagulation alone (12.8%/20.7%) [7] or other advanced therapies (11.8%/11.5%) [8]. Given the rapid growth in intermediate- and high-risk PE patients undergoing CDT and MT, anesthesia providers need to be aware of the specific risks these patients encounter to ensure optimal anesthesia care and patient safety.

Non-operating room anesthesia (NORA) presents several challenges to anesthesiologists, including radiation exposure, lack of operating room resources, ergonomic challenges, and unfamiliar procedures and personnel [9]. In particular, procedures in the cardiac catheterization laboratory and interventional radiology suites (such as CDT and MT) are associated with higher morbidity and mortality compared not only to other NORA locations but also to surgical operating rooms [10]. In addition to the usual risks of bleeding and reactions to intravenous contrast, atypical but not uncommon risks of MT include cardiovascular collapse from acute right heart failure. Therefore, anesthesia providers must be prepared to evaluate, resuscitate, and escalate therapy for these patients as necessary. The objective of this review article is to summarize the recent literature on CDT and MT to help inform anesthesia providers who are caring for patients undergoing these procedures outside of the operating room environment.

## 2. Emerging Percutaneous Procedures for Acute PE Treatment

The American Heart Association (AHA) classifies PE into three mortality risk groups: low risk, submassive, and massive [11]. The European Society of Cardiology (ESC) classifies early mortality risk into low, intermediate (with subcategories intermediate–low and intermediate–high), and high risk [12]. Submassive and intermediate-risk PE are defined by hemodynamic stability but exhibit evidence of RV injury or dysfunction, identified through either a CT scan or echocardiographic parameters. Submassive PE accounts for 40% of PEs, with a 30-day mortality rate ranging from 5 to 25% [13]. Massive or high-mortality-risk PEs are characterized by being hemodynamically unstable, often requiring vasoactive and/or inotropic medication support, with a 30-day mortality upward of 50–70% [14,15]. The current guidelines for both the AHA and ESC recommend treatment with anticoagulation (AC) for those with submassive and intermediate-risk PEs. Guidelines recommend tissue plasminogen activator (tPA) for massive PE and those with submassive or intermediate-risk PE who have clinical and hemodynamic deterioration. However, if there is a contraindication to tPA, catheter-directed or surgical therapy should be considered [11,12]. For patients with massive PE and those with submassive or intermediate-risk PE approaching hemodynamic instability, several catheter-directed devices have been developed. Two primary catheter-directed approaches include CDT and MT. Both CDT and MT are primarily performed in a cardiac catheterization lab or in the interventional radiology suite because of the need for specialized equipment and fluoroscopy.

The PINC AI™ Healthcare Database, which is a large US database, reported 426,194 hospitalizations for acute PE, with 7705 undergoing at least one catheter-directed therapy between January 2018 and March 2022 [16]. Medicare claims data from 2004 to 2016 showed that the treatment of PE with catheter-directed therapies had increased ten-fold [12]. The Agency for Healthcare Research and Quality’s nationwide inpatient sample, which is the largest all payer inpatient health care database in the US, evaluated admissions for PE over 2010 through 2018. They found 1,627,718 patients hospitalized for PE, and of those, 6531 underwent MT [17]. The above databases have revealed a rapid increase in CDT over the past decade [16,17,18].

## 3. Catheter-Directed Thrombolytic (CDT)

This technique utilizes a 5–7 French catheter placed by an interventional cardiologist or interventional radiologist through the femoral vein and advanced into the PE where tPA is infused through side holes near the tip of the catheter along with the application of high-frequency, low-power ultrasound energy into the clot, which assists in increasing the permeability of the tPA [19,20,21]. In the SEATTLE II trial, 119 patients with either massive or submassive PE underwent CDT, with a 30-day mortality of 2.5% and an incidence of 10% major bleeding without any ICH events [22]. In the KNOCOUT PE trial, 489 patients with either intermediate–high-risk or high-risk PE underwent CDT. The researchers reported a 30-day all-cause mortality rate of 1%, a 1.6% major bleeding rate, and no ICH events [23]. In a large meta-analysis by Ismayl et al., the researchers compared a total of 7918 patients with submassive PE who were treated with either CDT or AC. They found 30-day mortality rates of 2.4% in the CDT group and 10% in the AC group. Furthermore, they found similar rates of major bleeding (2.6% with CDT vs. 2.8% with AC) and similar rates of ICH (0.6% with CDT vs. 0.5% with AC) [24].

While CDT has shown promise with decreasing 30-day mortality compared to AC, there has been no randomized controlled trials (RCTs) comparing CDT to AC. Furthermore, no studies have looked at long-term outcomes following CDT compared with AC, such as rate of chronic thromboembolic pulmonary hypertension, chronic dyspnea, 1-year survival, 5-year survival, or recurrent PE rates.

## 4. Mechanical Thrombectomy (MT)

This technique utilizes a 16–24 French catheter placed either through the femoral vein or internal jugular vein by an interventional cardiologist or interventional radiologist and advanced into the PE. The interventionalist will then aspirate the thrombus directly from the pulmonary arteries. In the FLARE trial, 106 patients with submassive PE underwent MT using the FlowTreiver system (Inari Medical, Irvine, CA, USA). The researchers reported a 1% 30-day mortality rate, a 3.8% major adverse event rate with a 1% major bleeding rate, and no ICH events. The mode of anesthesia was local anesthesia in 96.2% of these patients. The average time to complete the procedure was 93.8 ± 29 min [25]. The FLASH trial utilized the second-generation FlowTreiver MT device and recruited 800 US patients with both high-risk and intermediate-risk PE. The researchers reported a 0.8% 30-day mortality rate, a 1.8% major adverse event rate with a 1.5% major bleeding rate, and no ICH events. This study also reported that 98.5% of the procedures were performed under local anesthesia or sedation and that three patients (0.4%) required ECMO cannulation. The average time to complete the procedure was 46 min [26]. The EXTRACT-PE study utilized the Indigo Aspiration MT system (Penumbra, Alameda, CA, USA) and recruited 119 patients with submassive PE. The researchers reported a 2.5% 30-day mortality rate, a 1.7% major adverse event rate and major bleeding rate, and no ICH events. The study reported that 97.5% of patients underwent conscious sedation, with a median procedure duration of 37 min [27]. The FLAME study was a prospective, multicenter, nonrandomized, parallel group, observational study of high-risk PE patients undergoing either MT utilizing the FlowTreiver (Inari Medical, Irvine, CA, USA) system vs. systemic tPA or AC alone. There were 53 patients that underwent MT, 42 patients received TPA, and 19 patients were treated with AC alone. The study reported a hospital mortality rate of 1.9% in the MT group compared to 29.5% in the tPA or AC alone groups, a major bleeding rate of 11.3% in the MT group compared to 24.6% in the tPA or AC alone groups, and no ICH events in the MT group compared to 3.3% in the tPA group. The study did not report average procedure times for the MT group, but 5.7% of the MT group required ECMO as part of their treatment compared to 11.5% in the tPA or AC groups [28].

There are potential complications that the anesthesia provider should be prepared to manage. MT catheters are large in diameter; with the advancement of these catheters into the PA, injury to the tricuspid or pulmonary valve may occur. These devices can also injure the PA, leading to massive hemoptysis and profound hemodynamic instability [26,27]. In addition, when blood is being aspirated from the PA, profound bradycardia as well as significant hemodynamic instability may ensue, as the right ventricle is relying on preload to maintain cardiac output to the left ventricle.

However, there are currently gaps in the literature with respect to MT. Like CDT, there is a lack of RCTs comparing MT to AC in the intermediate-risk PE population and tPA in the high-risk PE population. There is also a lack of long-term patient outcomes with MT, which is the same for CDT discussed above.

## 5. Institutional Protocol for PE Management

Anesthesia providers play a critical role in the assessment and management of PE patients undergoing interventional procedures. This responsibility includes risk stratification and active participation in multidisciplinary discussions within the Pulmonary Embolism Response Team (PERT) to ensure safe perioperative care.

The PERT model of care was established in 2012 to leverage expertise from multiple disciplines for timely clinical decision making in the management of complex acute PE [29]. This model has expanded both nationally and internationally, with over 100 centers operating in the United States alone [30]. While the composition of PERTs may vary by institution based on local expertise, teams typically include pulmonologists, interventionalists, critical care physicians, vascular medicine physicians, and cardiac surgeons [31]. PERT activation most frequently occurs in cases of intermediate or submassive PE, reflecting the limited data available to guide management and identify patients who may benefit from advanced interventions.

Our local protocol emphasizes the early involvement of PERT. When PERT is unavailable, close collaboration among anesthesia providers, interventionalists, critical care teams, and extra-corporeal membrane oxygenation (ECMO) specialists is essential. For centers without cardiac anesthesia or ECMO capabilities, interhospital transfer to a tertiary care facility with access to advanced therapies may be necessary.

Comprehensive patient stratification should be completed before any procedures, including a detailed history, clinical examination, review of perfusion markers, echocardiographic findings, and cardiac biomarkers. Special attention should be given to any signs or symptoms of malperfusion and shock. An echocardiographic assessment should include evaluations of right ventricular (RV) function and cardiac output/index. This approach ensures an accurate classification of patients in accordance with AHA and ESC guidelines and helps identify those at risk of deterioration [11,12].

For patients with massive or high-risk PE, an early consultation for ECMO initiation or prophylactic placement of venous and arterial sheaths is critical, provided there are no contraindications according to local ECMO guidelines. Additionally, the involvement of cardiac anesthesia providers is essential. General anesthesia (GA) should be avoided if possible due to the risk of hemodynamic compromise in the setting of right ventricular (RV) dysfunction [32]. Options such as high-flow nasal cannula or noninvasive positive pressure ventilation should be considered if hypoxia is a concern. However, if GA becomes necessary, the ECMO and procedural teams should be present and ready at the bedside in case of cardiovascular instability upon induction that requires immediate intervention. Careful titration of hemodynamically stable medications for GA induction, along with immediate availability of inotropic support such as epinephrine, milrinone, and pulmonary vasodilators like inhaled nitric oxide or inhaled prostanoids, is recommended [9]. Continuous electrocardiography monitoring is necessary due to the risk of arrhythmias or heart block during sheath placement and wire advancement. Invasive blood pressure monitoring is also necessary for close hemodynamic monitoring and prompt detection of any hemodynamic instability. Patients with pre-existing left heart block may be at risk for complete heart block, potentially requiring transcutaneous or transvenous pacing [9]. In addition, hemodynamic derangements with evidence of worsening cardiac output may necessitate the use of transthoracic echocardiography (usually in cases performed under monitored anesthesia care) or transesophageal echocardiography (usually in cases performed under general anesthesia) to assess right heart function [33].

Managing intermediate or submassive PEs presents its own challenges due to the limitations of most scoring systems in identifying patients at risk of deterioration [34]. As such, a vigilant approach similar to that used for massive PE is recommended. While ECMO initiation is typically unnecessary, special attention should be given to individuals with large, central clot burdens [35,36], saddle PEs with concomitant deep vein thrombosis (DVT) [37,38], subclinical or normotensive shock [34], clots in transit [39], and elevated lactate levels [40], as these patients are more vulnerable to deterioration and may progress to massive PE with further embolization.

Following the procedure, we recommend ICU admission for patients with massive PE, with consideration for floor admission for those with submassive PE, depending on the success of the procedure and hemodynamics.

## 6. Limitations and Future Directions

Percutaneous interventions for PE show promise but face limitations, including variable outcomes, potential industry bias, insufficient long-term data, and concerns about cost-effectiveness and complication reporting [41]. Ongoing advancements in device technology aim to enhance efficacy, streamline procedures, and incorporate hemodynamic monitoring. Nonetheless, large prospective studies remain essential to validate their long-term impact and establish their role in the management of PE.

## 7. Conclusions

Patients with PE present a distinct and complex risk profile, posing significant challenges for anesthesiologists during the perioperative period. As percutaneous procedures in NORA settings increase and technology advances, staying current with evolving techniques is vital. These trends highlight the need for meticulous periprocedural planning and early multidisciplinary engagement, with anesthesiologists playing a key role in managing complexities and optimizing patient outcomes.

## Data Availability

Not applicable.
